# Integrated bioinformatics analysis reveals marker genes and immune infiltration for pulmonary arterial hypertension

**DOI:** 10.1038/s41598-022-14307-6

**Published:** 2022-06-16

**Authors:** Shengxin Tang, Yue Liu, Bin Liu

**Affiliations:** 1 Department of Cardiovascular, The Third Affiliated Hospital of Chongqing Medical University, No. 1 Shuanghu Branch Road, Chongqing, 401120 China; 2 Department of Nursing, Youyoubaobei Hospital, Chongqing, 401122 China

**Keywords:** Cell biology, Computational biology and bioinformatics, Genetics, Molecular biology

## Abstract

Pulmonary arterial hypertension (PAH) is a chronic cardiopulmonary syndrome with high pulmonary vascular load and eventually causing RV heart failure even death. However, the mechanism of pulmonary hypertension remains unclear. The purpose of this research is to detect the underlying key genes and potential mechanism of PAH using several bioinformatic methods. The microarrays GSE22356, GSE131793 and GSE168905 were acquired from the GEO. Subsequently, a host of bioinformatics techniques such as DAVID, STRING, R language and Cytoscape were utilized to investigate DEGs between PAH and healthy controls and conduct GO annotation, KEGG enrichment analysis and PPI network construction etc. Additionally, we predicted the transcription factors regulating DEGs through iRegulon plugin of Cytoscape and CIBERSORT was used to conduct immune infiltration analysis. One thousand two hundred and seventy-seven DEGs (403 up-regulated and 874 down-regulated) were identified from peripheral blood samples of 32 PAH patients and 29 controls, among which SLC4A1, AHSP, ALAS2, CA1, HBD, SNCA, HBM, SELENBP1, SERPINE1 and ITGA2B were detected as hub genes. The functional enrichment changes of DEGs were mainly enriched in protein binding, extracellular exosome, extracellular space, extracellular region and integral component of plasma membrane. The hub genes are chiefly enriched at extracellular exosome, hemoglobin complex, blood microparticle, oxygen transporter activity. Among TF-DEGs network, 42 target DEGs and 6 TFs were predicted with an NES > 4 (TEAD4, TGIF2LY, GATA5, GATA1, GATA2, FOS). Immune infiltration analysis showed that monocytes occupied the largest proportion of immune cells. The trend analysis results of infiltration immune cells illustrated that PAH patients had higher infiltration of NK cell activation, monocyte, T cell CD4 memory activation, and mast cell than healthy controls and lower infiltration of T cell CD4 naive. We detected SLC4A1, AHSP, ALAS2, CA1, HBD, SNCA, HBM, SELENBP1, SERPINE1 and ITGA2B as the most significant markers of PAH. The PAH patients had higher infiltration of NK cell activation, monocyte, T cell CD4 memory activation, and mast cell than healthy controls and lower infiltration of T cell CD4 naive. These identified genes and these immune cells probably have precise regulatory relationships in the development of PAH.

## Introduction

Pulmonary arterial hypertension (PAH) is a chronic cardiopulmonary syndrome characterized by salient pulmonary vascular remodeling and accelerating increase in pulmonary vascular load, bringing about right ventricular (RV) enlargement and pulmonary blood vessels remodeling^1^ and ultimately leading to heart failure or even death if untreated^2^. Plentiful widespread cardiopulmonary diseases complicated with PAH, which badly increases morbidity and mortality^1^. PAH is a rare disease with reported prevalence of 15–50 cases in a million people and incidence of 5–10 cases in a million people every year^3^. Debate on the best strategy for PHA management continues despite great progress in treating PAH in recent decades. Extensive researched have shown current therapeutic approaches in PAH included prostacyclin receptor agonists, phosphodiesterase-5 (PDE-5) inhibitors, endothelin receptor antagonists (ERAs) and a soluble guanylate cyclase activator targeting several crucial signaling pathways that predominantly regulate pulmonary vasculature^4^. However, PAH may also can be caused by many unknown causes, such as pulmonary vascular remodeling and inflammation, which cannot be well solved by current drug treatment and PAH is still a complicated incurable cardiopulmonary disease^5^. Thus, it is necessary for us to utilize bioinformatics technology to explore the pathogenesis or potential treatments of PAH.

**Figure 1:**
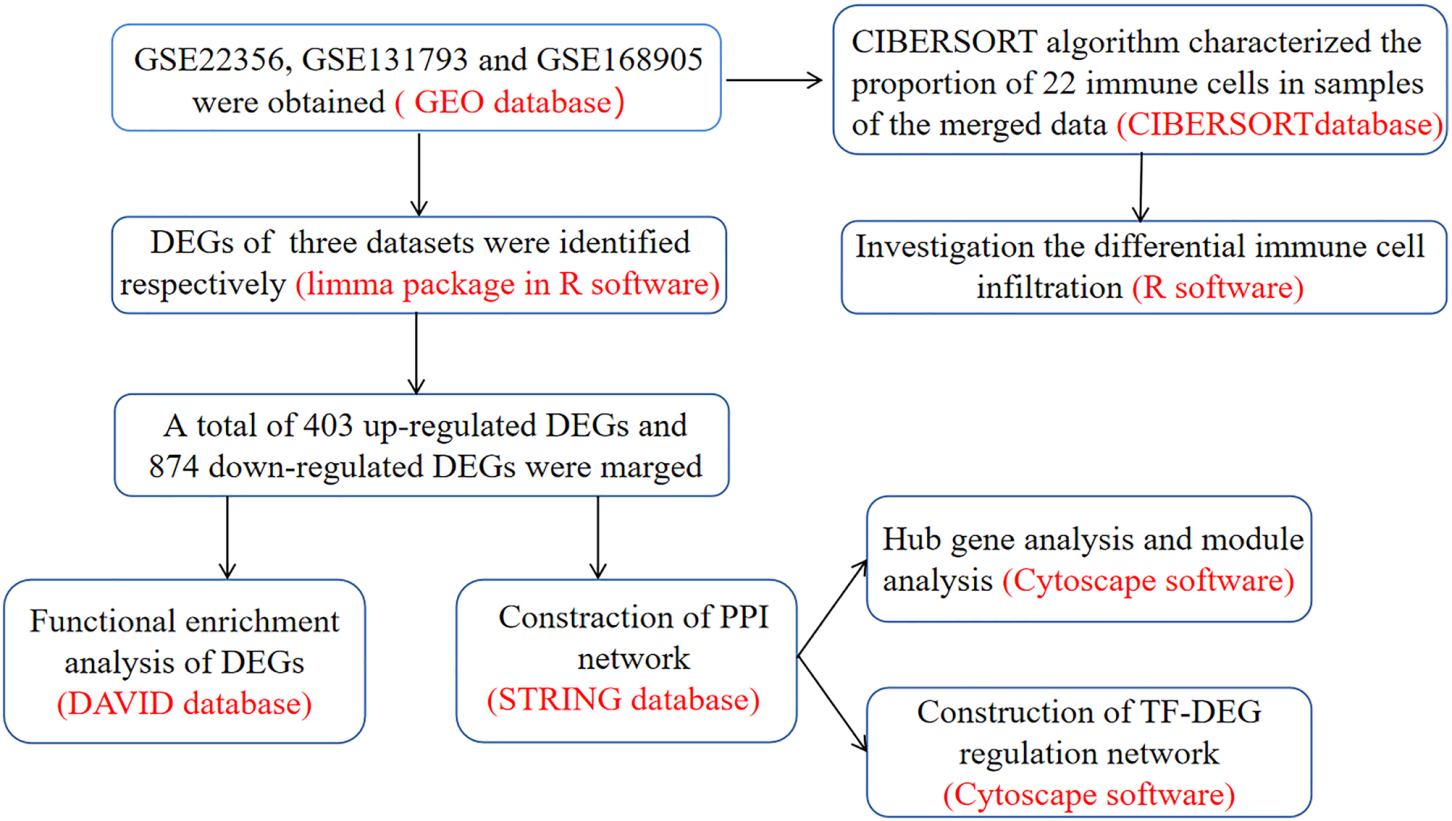
Flow chart of the analysis program in this study.

At present, bioinformatics methods have been proverbially performed to analyze gene sequencing data of various diseases to ascertain differentially expressed genes (DEGs) and implement various analyses. And more and more robust databases and powerful online tools have been established to help our repositioning of known intricate mechanism of diseases^6^. Increasing researches applied microarray technology for the purpose of searching DEGs and their molecular functions (MFs), biological processes (BPs), or cellular components (CCs) as well as relatedregulatory pathways in specific disease state^7^. For example, Habib Rahman and his colleagues used bioinformatics and machine learning methods to determine new factors that improve the identification and characterization of glioblastoma tumors and their progression^8^. Based on a neighborhood-based benchmarking and multilayer network topology techniques, they also identified novel putative biomarkers which manifest how type 2 diabetes (T2D) interact^9^. In our study, as the flow chart shown in Fig. 1, the expression datasets GSE22356, GSE131793 and GSE168905 were analyzed to identify key genes employing comprehensive bioinformatics analysis technologies, consisting of GO and KEGG analysis, protein–protein interaction (PPI) network construction, immune infiltration analysis and hub gene identification. Relying on the GEO database and R language, the distinguishing infiltration of 22 immune cells in peripheral blood of PAH patients were compared with healthy cases. The study probably revealed the pathogenic mechanism and potential therapeutic target of PAH.

## Results

### Identification of DEGs in PAH

**Figure 2:**
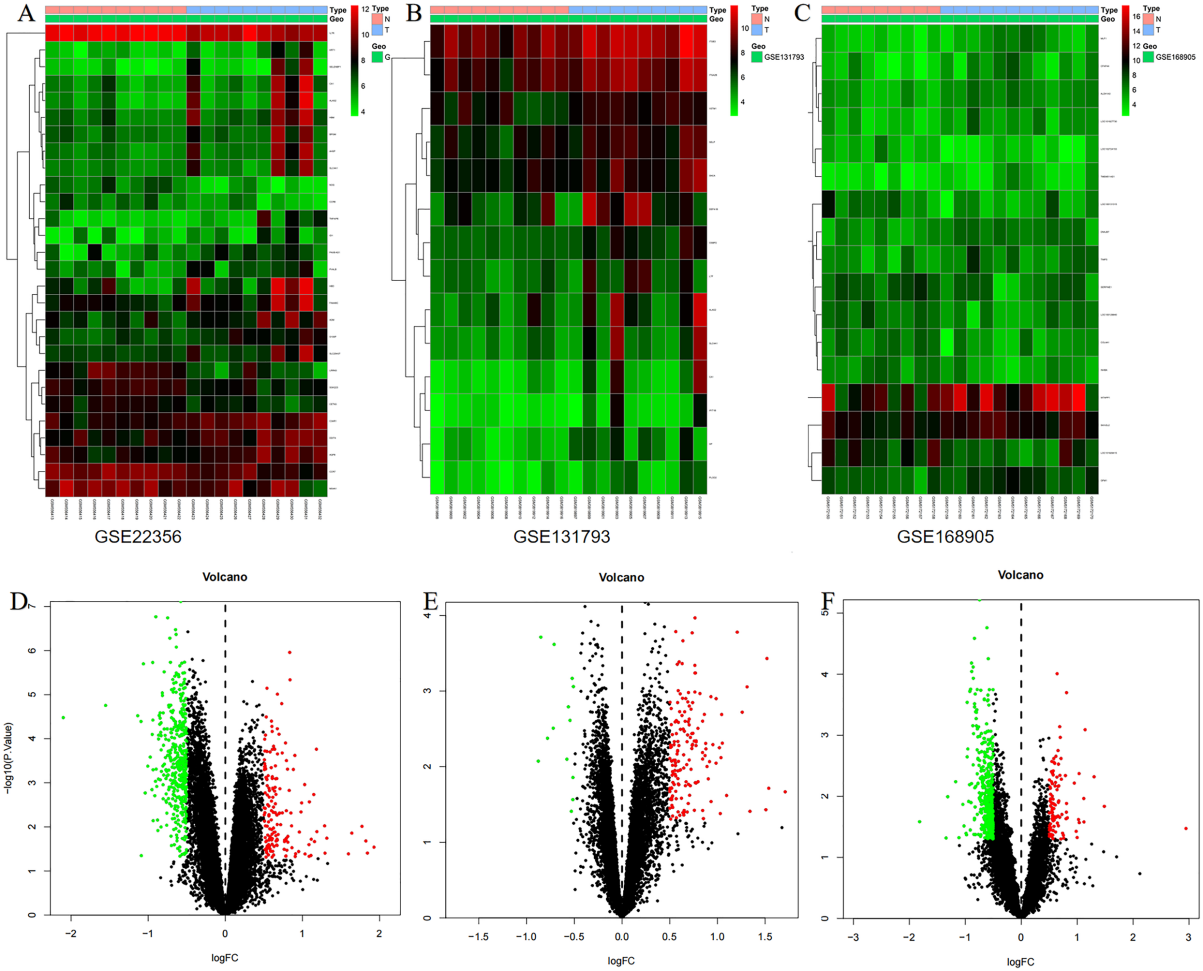
The identification of EDGs. (**A**–**C**) The heat-maps of differential expression genes of GSE22356, GSE131793 and GSE168905 (P < 0.05, |log FC|> 1). Up-regulated genes were in red and down-regulated genes were in blue. (**D**–**F**) Volcano plot of genes identified in PAH of GSE22356, GSE131793 and GSE168905 (P < 0.05, |log FC|> 0.5). Red dots represent up-regulated and green dots represent down-regulated.

There are 580 DEGs screened from GSE22356 between PAH group with healthy group which consists of 162 up-regulated and 418 down-regulated genes (Fig. 2A,D). Simultaneously, heatmaps and volcano plots vividly showed that 156 up-regulated and 13 down-regulated DEGs were identified from GSE131793, 102 up-regulated and 456 down-regulated DEGs were identified from GSE168905 (Fig. 2B,C,E,F) (p value < 0.05 and |log2FC|≥ 0.5). Then, we merged up- regulated and down-regulated DEGs in the three datasets, which revealed a total of 403 up-regulated DEGs and 874 down-regulated DEGs in the microarrays.

### Functional enrichment analysis of DEGs

**Figure 3:**
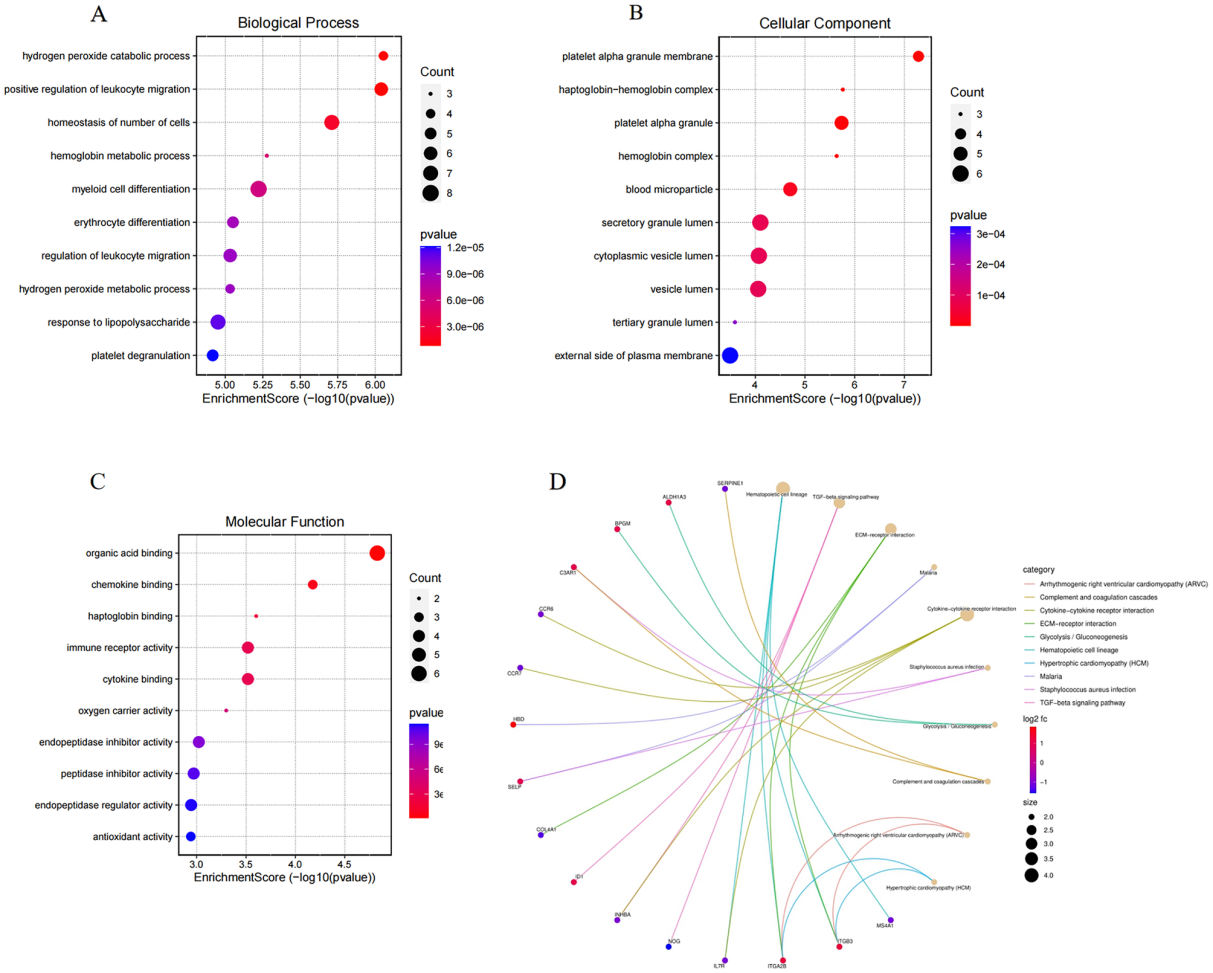
GO and KEGG analysis of DEGs between PAH and healthy groups. (**A**–**C**) The top 10 GO annotation results of DEGs. (**D**) KEGG pathway analyses of DEGs (top 10 according to enrichment score).

For demonstrating PAH‐related functional annotation and pathway enrichment, these DEGs with an absolute value of log2 fold change greater than 1 were selected for GO and KEGG analysis and detailed results are presented in Tables 1 and 2. In GO analysis, the richest BP participated hydrogen peroxide catabolic process, positive regulation of leukocyte migration and homeostasis of number of cells (Fig. 3A). In the CC category, platelet alpha granule membrane, haptoglobin-hemoglobin complex and platelet alpha granule were mostly interrelated with PAH (Fig. 3B). Organic acid binding, chemokine binding and haptoglobin binding were most obviously enriched for DEGs in the MF category (Fig. 3C). As we all know, KEGG mapping is a predictive method via reestablishing molecular network systems from molecular constructing blocks grounded on functional orthologs^10–12^. In KEGG analysis, the result indicated that genes were mainly associated with hematopoietic cell lineage, TGF-beta signaling pathway and ECM-receptor interaction (Fig. 3D).

**Table 1:** The top 10 BPs, CCs, MFs of GO functional annotation analyses of DEGs.

Term	Count	Gene ID	p. adjust
**GO-BPs**			
GO:0030099 ~ myeloid cell differentiation	8	SLC4A1/LTF/ITGA2B/INHBA/CCR7/BPGM/ALAS2/AHSP	0.001688296
GO:0048872 ~ homeostasis of number of cells	7	SLC4A1/INHBA/IL7R/CCR7/BPGM/ALAS2/AHSP	0.001033927
GO:0032496 ~ response to lipopolysaccharide	7	SNCA/SERPINE1/SELP/LTF/DEFA1B/CCR7/ADM	0.001688296
GO:0007596 ~ blood coagulation	7	SLC4A1/SERPINE1/SELP/KRT1/ITGB3/ITGA2B/HBD	0.001688296
GO:0007599 ~ hemostasis	7	SLC4A1/SERPINE1/SELP/KRT1/ITGB3/ITGA2B/HBD	0.001688296
GO:0050817 ~ coagulation	7	SLC4A1/SERPINE1/SELP/KRT1/ITGB3/ITGA2B/HBD	0.001688296
GO:0002237 ~ response to molecule of bacterial origin	7	SNCA/SERPINE1/SELP/LTF/DEFA1B/CCR7/ADM	0.001791109
GO:0006959 ~ humoral immune response	7	MS4A1/LTF/KRT1/DEFA1B/CCR7/CCR6/C3AR1	0.002283905
GO:0043312 ~ neutrophil degranulation	7	TNFAIP6/S100P/LTF/KRT1/HP/DEFA1B/C3AR1	0.007504148
GO:0002283 ~ neutrophil activation involved in immune response	7	TNFAIP6/S100P/LTF/KRT1/HP/DEFA1B/C3AR1	0.007506536
**GO-CCs**			
GO:0034774 ~ secretory granule lumen	6	TIMP3/SERPINE1/S100P/LTF/HP/DEFA1B	0.001216318
GO:0060205 ~ cytoplasmic vesicle lumen	6	TIMP3/SERPINE1/S100P/LTF/HP/DEFA1B	0.001216318
GO:0031983 ~ vesicle lumen	6	TIMP3/SERPINE1/S100P/LTF/HP/DEFA1B	0.001216318
GO:0009897 ~ external side of plasma membrane	6	SELP/MS4A1/ITGA2B/IL7R/CCR7/CCR6	0.003575905
GO:0031091 ~ platelet alpha granule	5	SNCA/SERPINE1/SELP/ITGB3/ITGA2B	6.39342E-05
GO:0072562 ~ blood microparticle	5	SLC4A1/KRT1/ITGA2B/HP/HBD	0.000440622
GO:0030667 ~ secretory granule membrane	5	SNCA/SELP/ITGB3/ITGA2B/C3AR1	0.006030401
GO:0062023 ~ collagen-containing extracellular matrix	5	TIMP3/SERPINE1/KRT1/DEFA1B/COL4A1	0.018115583
GO:0031092 ~ platelet alpha granule membrane	4	SNCA/SELP/ITGB3/ITGA2B	5.75912E-06
GO:0031838 ~ haptoglobin-hemoglobin complex	3	HP/HBM/HBD	6.39342E-05
**GO-MFs**			
GO:0043177 ~ organic acid binding	6	TNFAIP6/SELP/PLOD2/HBM/HBD/ALAS2	0.002918932
GO:0019955 ~ cytokine binding	4	NOG/ITGB3/CCR7/CCR6	0.011923107
GO:0140375 ~ immune receptor activity	4	IL7R/CCR7/CCR6/C3AR1	0.011923107
GO:0004866 ~ endopeptidase inhibitor activity	4	TIMP3/SNCA/SERPINE1/LTF	0.021672983
GO:0030414 ~ peptidase inhibitor activity	4	TIMP3/SNCA/SERPINE1/LTF	0.021672983
GO:0061135 ~ endopeptidase regulator activity	4	TIMP3/SNCA/SERPINE1/LTF	0.021672983
GO:0031406 ~ carboxylic acid binding	4	TNFAIP6/SELP/PLOD2/ALAS2	0.021672983
GO:0061134 ~ peptidase regulator activity	4	TIMP3/SNCA/SERPINE1/LTF	0.025123719
GO:0004857 ~ enzyme inhibitor activity	4	TIMP3/SNCA/SERPINE1/LTF	0.095869699
GO:0019956 ~ chemokine binding	3	ITGB3/CCR7/CCR6	0.0065716

**Table 2:** The top 12 KEGG enrichment pathway analysis of DEGs.

Term	Count	Genes	P value
hsa04640 ~ Hematopoietic cell lineage	4	MS4A1/ITGB3/ITGA2B/IL7R	0.000392423
hsa04060 ~ Cytokine-cytokine receptor interaction	4	INHBA/IL7R/CCR7/CCR6	0.020856078
hsa04350 ~ TGF-beta signaling pathway	3	NOG/INHBA/ID1	0.004707872
hsa04512 ~ ECM-receptor interaction	3	ITGB3/ITGA2B/COL4A1	0.004707872
hsa04510 ~ Focal adhesion	3	ITGB3/ITGA2B/COL4A1	0.046127511
hsa05144 ~ Malaria	2	SELP/HBD	0.017943455
hsa05150 ~ Staphylococcus aureus infection	2	SELP/C3AR1	0.021407095
hsa00010 ~ Glycolysis / Gluconeogenesis	2	BPGM/ALDH1A3	0.028279271
hsa04610 ~ Complement and coagulation cascades	2	SERPINE1/C3AR1	0.031583311
hsa05412 ~ Arrhythmogenic right ventricular cardiomyopathy (ARVC)	2	ITGB3/ITGA2B	0.035918255
hsa05410 ~ Hypertrophic cardiomyopathy (HCM)	2	ITGB3/ITGA2B	0.044264599
hsa05222 ~ Small cell lung cancer	2	ITGA2B/COL4A1	0.046209667

### PPI network construction and hub genes identification

**Figure 4:**
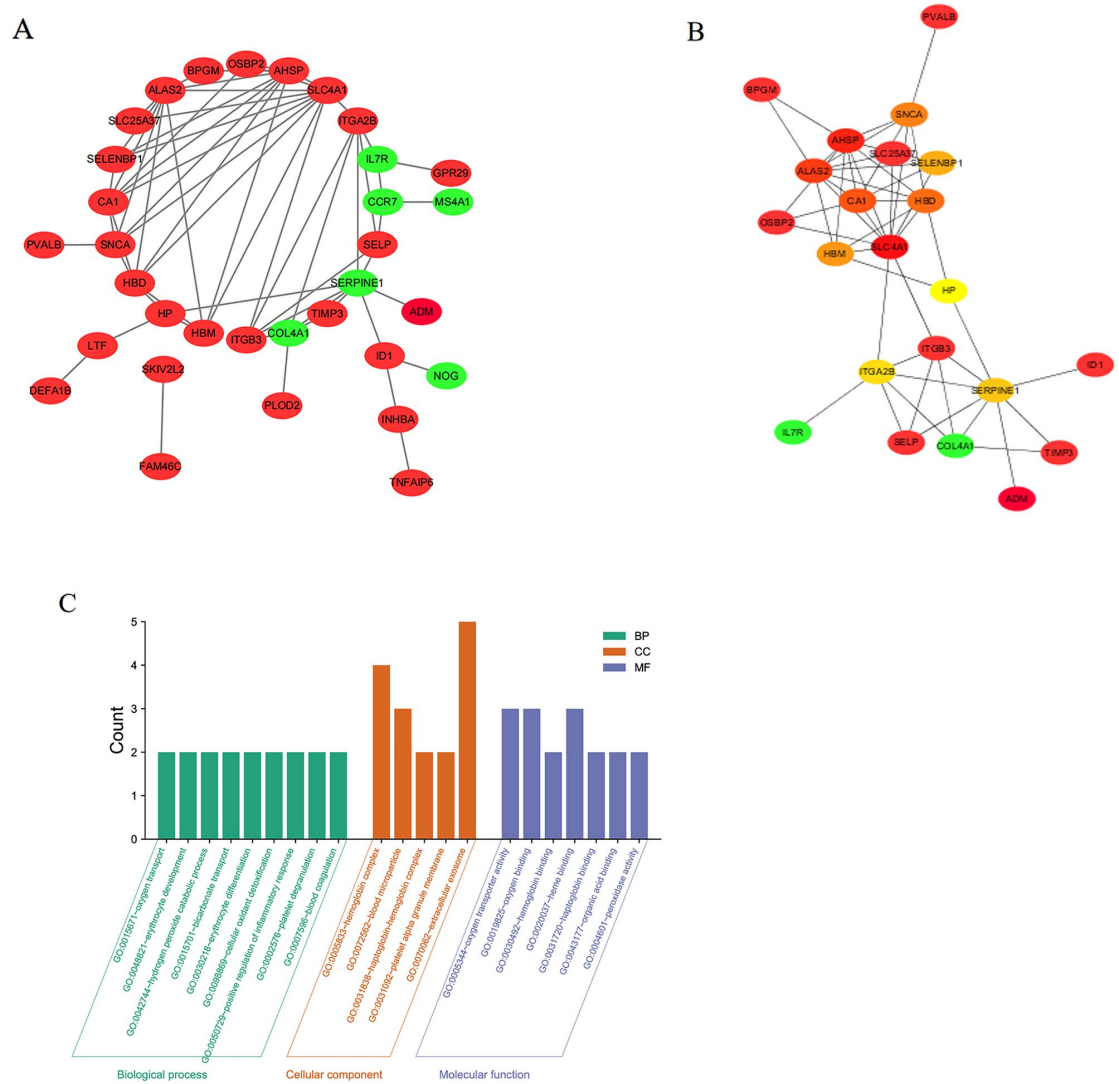
(**A**) The PPI network of DEGs was established using string and Cytoscape, containing 33 nodes and 57 edges. The upregulated genes are red and downregulated are green. (**B**) The top 10 hub genes were detected from the PPI network. (**C**) GO terms of the top 10 key genes.

Uploading 47 DEGs into the STRING database, a PPI network with the desired interaction score > 0.48 was constructed and the network containing 33 nodes and 57 edges was visualized via Cytoscape software (Fig. 4A). Then the most remarkable module was recognized by cytoHubba plug-in according to the maximal clique centrality topology analysis methods (Fig. 4B). The top 10 significant nodes with the highest connectivity among the network were appraised as hub genes: SLC4A1, AHSP, ALAS2, CA1, HBD, SNCA, HBM, SELENBP1, SERPINE1, ITGA2B (Table 3). Additionally, the most significantly enriched BPs showed that hub genes were related to oxygen transport, erythrocyte development, hydrogen peroxide catabolic process bicarbonate transport. The changes of CCs containing hemoglobin complex, blood microparticle, haptoglobin-hemoglobin complex and platelet alpha granule membrane. MFs were mainly enriched in hemoglobin binding, haptoglobin binding, oxygen transporter activity and organic acid binding (Fig. 4C).

**Table 3:** Summary of the function of 10 key genes.

Gene symbol	Full name	Function
SLC4A1	Solute carrier family 4 member 1	PART of the anion exchanger (AE) family, expressed in the erythrocyte plasma membrane, where it functions as a chloride/bicarbonate exchanger involved in carbon dioxide transport from tissues to lungs;This protein is predominantly dimeric but forms tetramers in the presence of ankyrin. Many mutations in this gene are known in man, and these mutations can lead to two types of disease: destabilization of red cell membrane leading to hereditary spherocytosis, and defective kidney acid secretion leading to distal renal tubular acidosis
AHSP	Alpha hemoglobin stabilizing protein	Encodes a molecular chaperone which binds specifically to free alpha-globin and is involved in hemoglobin assembly. The encoded protein binds to monomeric alpha-globin until it has been transferred to beta-globin to form a heterodimer, which in turn binds to another heterodimer to form the stable tetrameric hemoglobin. Diseases associated with AHSP include Beta-Thalassemia and Thalassemia. GO annotations related to this gene include unfolded protein binding and hemoglobin binding. Acts as a chaperone to prevent the harmful aggregation of alpha-hemoglobin during normal erythroid cell development. Specifically protects free alpha-hemoglobin from precipitation
ALAS2	5′-Aminolevulinate synthase 2	An erythrocyte-specific mitochondrial localization enzyme and that step one of the heme biosynthetic pathway is catalyzed by this product; Blemishes on ALAS2 will lead to the development of X-linked pyridoxine-responsive sideroblastic anemia.Diseases associated with ALAS2 include Anemia, Sideroblastic, 1 and Protoporphyria, Erythropoietic, X-Linked. Among its related pathways are Metabolism and Metabolism of porphyrins. Gene Ontology (GO) annotations related to this gene include pyridoxal phosphate binding and glycine binding
CA1	Carbonic anhydrase 1	Carbonic anhydrases (CAs) are a large family of zinc metalloenzymes that catalyze the reversible hydration of carbon dioxide. They participate in a variety of biological processes, including respiration, calcification, acid–base balance, bone resorption, and the formation of aqueous humor, cerebrospinal fluid, saliva and gastric acid. They show extensive diversity in tissue distribution and in their subcellular localization. This CA1 gene is closely linked to the CA2 and CA3 genes on chromosome 8. It encodes a cytosolic protein that is found at the highest level in erythrocytes. Diseases associated with CA1 include Subacute Thyroiditis and Transient Global Amnesia. Among its related pathways are Metabolism and Reversible hydration of carbon dioxide. Gene Ontology (GO) annotations related to this gene include carbonate dehydratase activity and hydro-lyase activity
HBD	Hemoglobin subunit delta	The delta (HBD) gene is normally expressed in the adult, Two alpha chains plus two delta chains constitute HbA-2, which with HbF comprises the adult hemoglobin. Mutations in the delta-globin gene are associated with hemoglobin lepore-beta-thalassemia syndrome and fetal hemoglobin quantitative trait locus1. Among its related pathways are Factors involved in megakaryocyte development and platelet production and Response to elevated platelet cytosolic Ca2 + . Gene Ontology (GO) annotations related to this gene include iron ion binding and oxygen binding
SNCA	Synuclein alpha	a member of the synuclein family, which also includes beta- and gamma-synuclein. Synucleins are abundantly expressed in the brain and alpha- and beta-synuclein inhibit phospholipase D2 selectively. serve to integrate presynaptic signaling and membrane trafficking. SNCA peptides are a major component of amyloid plaques in the brains of patients with Alzheimer's disease. Alternatively spliced transcripts encoding different isoforms have been identified for this gene. Diseases associated with SNCA include Dementia, Lewy Body and Parkinson Disease 1, Autosomal Dominant. Gene Ontology (GO) annotations related to this gene include calcium ion binding and enzyme binding
HBM	Hemoglobin subunit Mu	HBM gene has an ORF encoding a 141 aa polypeptide which is similar to the delta globins found in reptiles and birds. Diseases associated with HBM include Alcohol-Induced Mental Disorder and Alcoholic Psychosis. Gene Ontology (GO) annotations related to this gene include iron ion binding and oxygen binding
SELENBP1	Serpin family E member 1	A member of the selenium-binding protein family. Selenium is an essential nutrient that exhibits potent anticarcinogenic properties, and deficiency of selenium may cause certain neurologic diseases. The effects of selenium in preventing cancer and neurologic diseases may be mediated by selenium-binding proteins, and decreased expression of this gene may be associated with several types of cancer. The encoded protein may play a selenium-dependent role in ubiquitination/ deubiquitination-mediated protein degradation
SERPINE1	Serpin family E member 1	A member of the serine proteinase inhibitor (serpin) superfamily. This member is the principal inhibitor of tissue plasminogen activator (tPA) and urokinase (uPA), and hence is an inhibitor of fibrinolysis. The protein also functions as a component of innate antiviral immunity. Defects in this gene are the cause of plasminogen activator inhibitor-1 deficiency (PAI-1 deficiency), and high concentrations of the gene product are associated with thrombophilia. Among its related pathways are G-protein signaling Ras family GTPases in kinase cascades (scheme) and hypothesized pathways in pathogenesis of cardiovascular disease. GO annotations related to this gene include signaling receptor binding and protease binding
ITGA2B	Pro-platelet basic protein	a member of the integrin alpha chain family of proteins. The encoded preproprotein is proteolytically processed to generate light and heavy chains that associate through disulfide linkages to form a subunit of the alpha-IIb/beta-3 integrin cell adhesion receptor. This receptor plays a crucial role in the blood coagulation system, by mediating platelet aggregation. Mutations in this gene are associated with platelet-type bleeding disorders, which are characterized by a failure of platelet aggregation, including Glanzmann thrombasthenia

### Analysis of TF-target regulating networks

**Figure 5:**
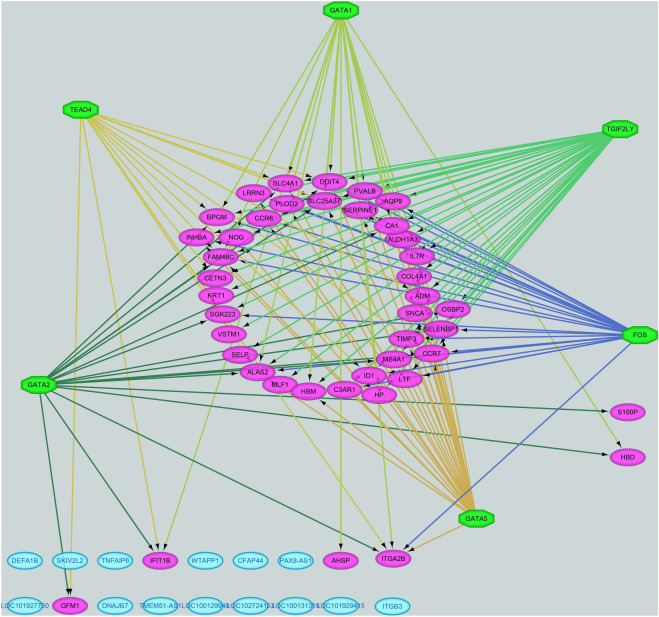
The TF-DEGs network including 42 DEGs and 6 TFs with an NES > 4.

To gain further insight into the regulatory TFs and selected DEGs, we used the iRegulon plugin to predict the TFs of DEGs. There were 48 nodes were predicted consisting of 42 DEGs and 6 TFs with an NES > 4 in this TF-DEGs network (TEAD4, TGIF2LY, GATA5, GATA1, GATA2, FOS) (Fig. 5). Specifically, the results show that 36 DEGs were forecasted as targets of TGIF2LY, 15 DEGs were forecasted as targets of TEAD4, GATA2 is predicted to have 17 DEG targets, GATA5 has 25 DEG targets, GATA1 has19 DEGs, and FOS has 23 DEGs^13^. Three predicted TFs belong to the family of GATA zinc finger TFs that bind to various motifs in the promoters of DEGs. It can be speculated that the TFs regulates the development and progression of PAH by activating or repressing the transcription of DEGs (details of transcription factors are shown in Table 4).

**Table 4:** Summary of the function of 6 TFs.

Transcription factor	Full name	Function
TEAD4	TEA domain transcription factor 4	a member of the transcriptional enhancer factor (TEF) family of transcription factors, which contain the TEA/ATTS DNA-binding domain. It is preferentially expressed in the skeletal muscle, and binds to the M-CAT regulatory element found in promoters of muscle-specific genes to direct their gene expression. Alternatively spliced transcripts encoding distinct isoforms, some of which are translated through the use of a non-AUG (UUG) initiation codon
TGIF2LY	TGFB induced factor homeobox 2 like y-linked	a member of the TALE/TGIF homeobox family of transcription factors. This gene lies within the male specific region of chromosome Y, in a block of sequence that is thought to be the result of a large X-to-Y transposition. The C-terminus of this protein is divergent from that of its chromosome X homolog (TGIF2LX), suggesting that this protein may act as a regulator of TGIF2LX
GATA5	GATA binding protein 5	a transcription factor that contains two GATA-type zinc fingers. The encoded protein is known to bind to hepatocyte nuclear factor-1alpha (HNF-1alpha), and this interaction is essential for cooperative activation of the intestinal lactase-phlorizin hydrolase promoter. In other organisms, similar proteins may be involved in the establishment of cardiac smooth muscle cell diversity
GATA1	GATA binding protein 1	Transcriptional activator or repressor which probably serves as a general switch factor for erythroid development. It binds to DNA sites with the consensus sequence 5'-[AT]GATA[AG]-3' within regulatory regions of globin genes and of other genes expressed in erythroid cells. Activates the transcription of genes involved in erythroid differentiation of K562 erythroleukemia cells, including HBB, HBG1/2, ALAS2 and HMBS
GATA2	GATA binding protein 2	a transcription factor involved in stem cell maintenance with key roles in hematopoietic development. GATA2 mutations are associated with a variety of inherited and acquired immune disorders including myelodysplastic syndrome and acute myeloid leukemia. In addition to a role in hematopoiesis, the maintenance GATA2 expression has been implicated as a requirement in KRAS-driven non-small cell lung cancer. Preclinical models have indicated therapeutic benefit from targeting GATA2-mediated pathways in the context of KRAS-driven NSCLC
FOS	Fos proto-oncogene	The Fos gene family consists of 4 members: FOS, FOSB, FOSL1, and FOSL2. These genes encode leucine zipper proteins that can dimerize with proteins of the JUN family, thereby forming the transcription factor complex AP-1. As such, the FOS proteins have been implicated as regulators of cell proliferation, differentiation, and transformation. In some cases, expression of the FOS gene has also been associated with apoptotic cell death

### Immune cells infiltration analysis

**Figure 6:**
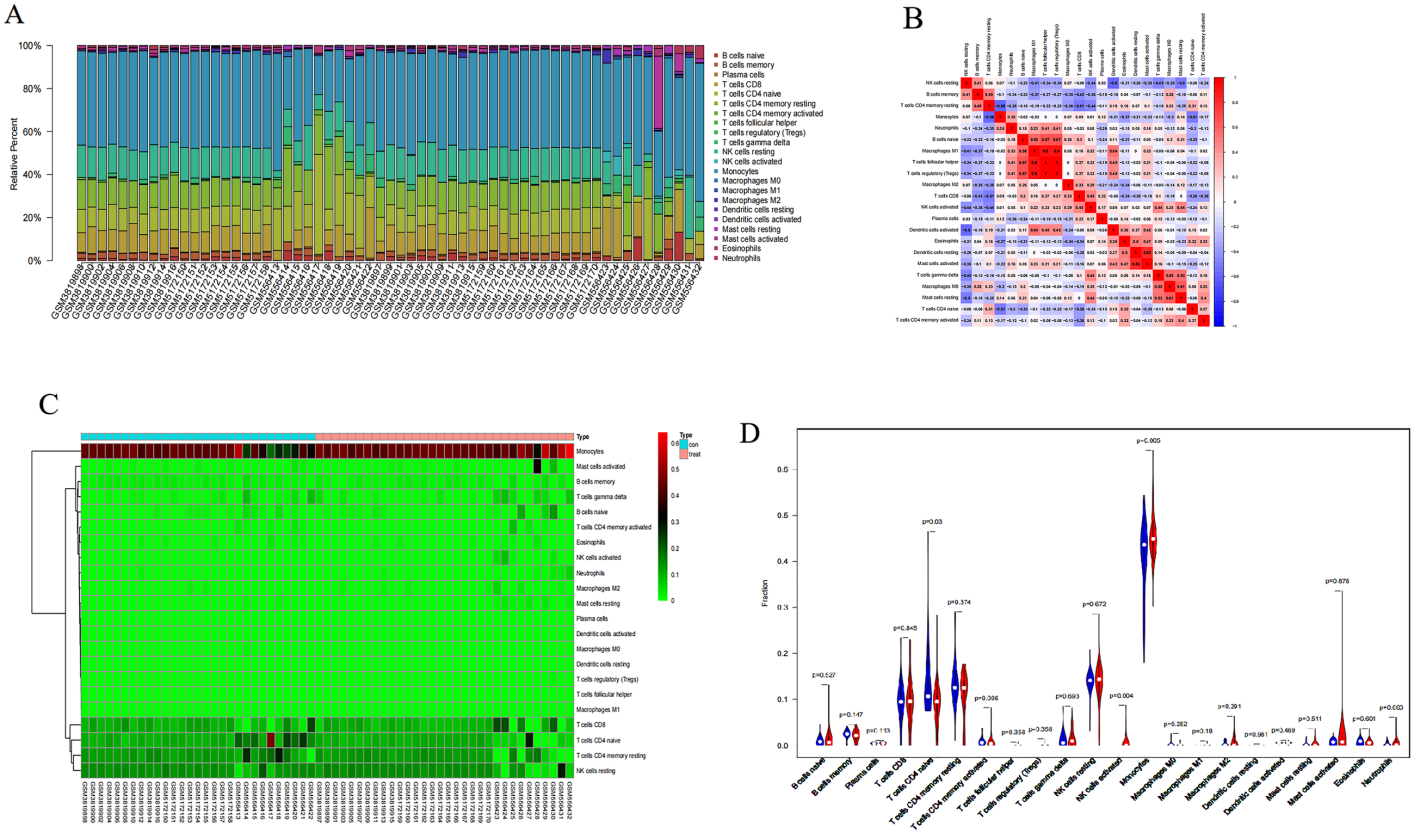
Summary of immune cell subpopulations between normal controls and PAH. (**A**) Composition of 22 kinds of immune cells subsets from GSE22356, GSE131793 and GSE168905 datasets. (**B**) Heatmap shows differences between the 22 subpopulations cell types and samples. (**C**) Correlation matrix of the 22 immunocyte proportions in healthy control and PAH samples. Red colors represent positive and blue colors represent negative correlations. (**D**) The difference of immune cells infiltration between 9 normal controls and 12 PAH (blue color represent normal controls group and red colors represent PAH group. P values < 0.05).

Using CIBERSORT, the relative proportions of 22 infiltration immunocytes in PAH and control samples were determined (Fig. 6A) and details were presented in Table 3. Furthermore, the heatmap showed that the 22 subpopulations of immunocytes were significantly different between PAH and controls and it was found that monocytes occupied the largest proportion of immune cells (Fig. 6B). Correlation analysis discovered that macrophages M had the most obvious positive relationship with both T cells follicular helper and T cells regulatory (Tregs) (r = 0.9, r = 0.9), dendritic cells resting and mast cells activated were also highly positively correlated with r = 0.83, while the strongest negative correlation was between dendritic cell activation and NK cell quiescence with r = − 0.6 (Fig. 6C). And the trend analysis results of infiltration immune cells illustrated that PAH patients had higher infiltration of NK cell activation, monocyte, T cell CD4 memory activation, and mast cell than healthy controls and lower infiltration of T cell CD4 naive (Fig. 6D; p < 0.05).

## Discussion

PAH is a rare chronic refractory syndrome accompanied with pathological changes of distal pulmonary arteries such as vasculature remodeling, lumen stenosis, laminar intimal proliferation and fibrosis, plexiform lesions with excessive poorly formed capillaries^14^. The current clinical definition of PAH is that the average pulmonary arterial pressure (m PAP) ≥ 25 mm Hg measured during right heart catheterization (1 mmHg = 0.133 kPa)^15^. PAH is divided into many subgroups due to different etiologies worldwide. According to the latest Chinese PAH registry, coronary heart disease-related PAH was reported as the most prevalent subtype, accounting for about 43% of all cases^3^. In addition to anticoagulant, calcium channel blockers^16^, diuretics, exercise rehabilitation^17^, oxygen therapy^18^ and other basic measures and conventional therapies to reduce right ventricular preload and improve left ventricular filling, the treatment of PAH also includes drugs targeting the prostacyclin pathway, endothelin pathway, nitric oxide-cGMP pathway like epoprostenol, iloprost, treprostinil, bosentan, ambrisentan, cediline, nafil, sildenafil, riociguat etc.^5,19^. Previous study has reported that tyrosine kinase inhibition (TKI) has an inhibitory effect on cell proliferation against pulmonary vascular remodeling^20^. Despite substantial efforts were made in PAH field in recent years, the pathogenesis of PAH remains to be further elucidated. Nearly decades, rapid advances in gene sequencing technologies and bioinformatics related science have made it possible to utilize abundant sequencing data for further analyzing. In this research, we identified 403 up-regulated and 874 down-regulated DEGs from peripheral blood samples belong to 29 controls and 32 PAH patients whose expression profiles downloaded via GEO database. Besides, PPI network construction and functional enrichment analyses were performed to filter key genes and important pathways. GO annotation results manifested that DEGs were enriched in extracellular exosome, integral component of plasma membrane, platelet degranulation, blood microparticle, extracellular space, extracellular region, immune response, cell surface and protein binding. KEGG pathway results declared that DEGs were mainly mapped to hematopoietic cell lineage, cytokine-cytokine receptor interaction, transcription growth factor beta (TGF-β) signaling pathway, extracellular mechanism (ECM) receptor interaction and focal adhesion.

Establishing PPI network is friendly for researchers to investigate the underlying molecular mechanism of PAH for the reason that the DEGs would be grouped and ordered in the network judging by their interactions^21^. Of which, 10 hub genes (SLC4A1, AHSP, ALAS2, CA1, HBD, SNCA, HBM, SELENBP1, SERPINE1, ITGA2B) related to PAH were detected owing to Cytoscape. It is speculated that these significant DEGs theoretically lead to the occurrence and progression of PAH. SLC4A1, solute carrier family 4 member 1, a member of the anion exchanger (AE) family, expressed mainly in the erythrocyte plasma membrane, functions as a chloride/bicarbonate exchanger in the erythrocyte plasma membrane associated with the transport of carbon dioxide from tissues to the lungs^22^. Recently a genome-wide association study revealed that candidate genes including SLC4A1 known to regulate structure of erythrocyte, metabolic process and ion channels^23^. Kaneda et al. used a lung cancer (LC) model mice to examine the proteome of cancer cell-secreted extracellular vesicles (cEVs) and found that SLC4A1 was co-expressed in CHL1-expressing EVs^24^. AHSP, Alpha-hemoglobin-stabilizing protein, is reported as a molecular chaperone, which peculiarly attaches to free α-globin and participates in hemoglobin assembly. This protein links to monomeric alpha-globin while it is transferred to beta-globin to compose a heterodimer, which then conversely unites to another heterodimer to shape a sturdy tetrameric hemoglobin^25^. During normal erythroid development, this protein functions as a chaperone to guard against deleterious aggregation of alpha-hemoglobin, particularly, to defend free alpha-hemoglobin from precipitation. AHSP regulates pathological conditions with excess alpha-hemoglobin like beta-thalassemia^26^. Protein 5′-Aminolevulinate Synthase 2 of ALAS2 is an erythrocyte-specific mitochondrial localization enzyme and that step one of the heme biosynthetic pathway is catalyzed by this product^27^. Blemishes on ALAS2 will lead to the development of X-linked pyridoxine-responsive sideroblastic anemia^28^.

The evidence presented thus far supports the idea that carbonic anhydrases (CAs) are zinc metalloenzymes mainly involved in catalyzing the reversible hydration of carbon dioxide^29^. CAs are involved in diverse biological processes, consisting of respiration, calcification, acid–base balance, bone resorption and so on. Studies have confirmed that CA1 gene is tightly related to the CA2 and CA3 genes on chromosome 8 and encrypts the highest levels of cytosolic proteins found in red blood cells^30^. Previous studies said the HBD gene was normally expressed in adults and encodes a delta chain that two delta chains add two alpha chains make up HbA-2, then together with HbF makes up adult hemoglobin. The research to date has tended to display that mutation in the HBD gene give rise to hemoglobin leprosy-beta-thalassemia syndrome and fetal hemoglobin quantitative trait loci^31^. The HBM gene has an open reading frame (ORF) that enciphers a 141 amino acid polypeptide resemble the delta globin unearthed in reptiles and birds. HBD-related pathways include factors involved in megakaryocyte development and thrombopoiesis and responses to elevated platelet cytoplasmic Ca^2+^. Alpha-synuclein, encoded by SNCA, belongs to the synuclein family including beta- and gamma-synuclein as well. Synuclein is plentifully expressed in the cerebrum, α- and β-synuclein selectively inhibits phospholipase D2. Protein SNCA can be used to analyze and integrate presynaptic semaphore and membrane transportation. It has been reported that SNCA peptides are the governing elements of amyloid patches in the brains of victims suffering from Alzheimer^32^. SERPINE1 gene a serine protease that is classified to the serine protease inhibitor (serpin) superfamily and a major inhibitor for tissue plasminogen activator (tPA) and urokinase (uPA). In addition, it is capable of congenial antiviral immunity. Studies have shown that SERPINE1 deficiency is responsible for plasminogen activator inhibitor 1 deficiency (PAI-1), and the high consistence proteins linked to thrombophilia^33^. Selenium Binding Protein 1, encoded by SELENBP1, pertain to the selenium-binding protein family. As we all known selenium is an indispensable trace element that possesses formidable anticarcinogenic characters. Researchers have proved that lack of selenium probably results in some certain neurological diseases. The role of selenium in the precaution of carcinoma and neurological diseases perchance is regulated through selenium-united proteins, and alleviated expression of SELENBP1 presumably is relevant to a few kinds of cancer^34^. This protein might selenium-dependently function in degrading ubiquitination/deubiquitination-regulated protein. SELENBP1 relational disorders include Extraoral Halitosis due to methanethiol oxidase deficiency and methionine adenosyltransferase deficiency^35^. Integrin Subunit Alpha 2b (ITGA2B) is geared to integrin alpha chain proteins family. The preproprotein undergoes proteolytic processing to produce light and heavy chains that binding via disulfide bonds to constitute subunits of the alpha-IIb/beta-3 integrin cell adhesion receptor. This receptor plays a vital character in coagulation system via attracting platelet gathering. Platelet-type bleeding disorders such as Glanzmann thrombasthenia are caused by mutations in ITGA2B manifesting the inability of platelets to aggregate^36^.

Furthermore, our current analysis also identified some TFs (TEAD4, TGIF2LY, GATA5, GATA1, GATA2, FOS) related to PAH, insinuating that these DEGs serve an important function in PAH. Next, based on current relevant studies we take a closer look at the association between PAHs and identified TFs. TEAD4, TEA domain transcription factor 4, is important component in Hippo signaling pathway which is involved in controlling organ growth and inhibiting tumor through limiting cell proliferation and facilitating apoptosis. TEAD4 mediates cell proliferation, migration, and epithelial-mesenchymal transition (EMT) by mediating the gene expression of YAP1 and WWTR1/TAZ. Previous study showed that Luteolin ameliorates reconstitution of pulmonary blood vessels and RV hypertrophy on rats PAH model and inhibited smooth muscle cells proliferation and migration at a dose-dependent manner through inhibition mediated by HIPPO-YAP/PI3K/AKT pathway^37^. TGIF2LY, belongs to the TALE/TGIF homeobox family of transcription factors, is located in the specific region of chromosome Y, in a large stretch of sequence which is considered as a X-to-Y transposition. It is possible to exert transcriptional regulation in testis and become a competitor or regulator of gene TGIF2LX. There are no studies to date that TGIF2LY is associated with PAH yet. The GATA family of transcription factors members GATA1, GATA2and GATA5 are associated with the extent of amino acid sequence identity in DNA zinc finger binding domains and they all have the capability to link the GATA sequence. Considering reprogramming function of GATA1/2/5, the three GATA family members are inducers of pluripotency reprogramming. Specifically, as a transcriptional activator or repressor, GATA1 possibly play a general switch role during erythroid development. GATA1 combines to the DNA sites with the common sequence 5′-GATA-3′ inside modulatory districts of globin genes and others genes of red blood cells and motivates genes transcription concerned with erythroid differentiation of K562 erythroleukemia cells, containing HBB, HBG1/2, ALAS2 and HMBS. GATA2 is a transcription factor participating in stem cell sustain, hematopoiesis and mediating endothelin-1 gene expression of endothelial cells. GATA5 is an indispensable TF during cardiovascular development^38^ and transcriptional program, which is the basis of diversity of smooth muscle cell. It’s reported that GATA5 united in the CEF-1 nucleoprotein binding domain of cardiac-specific slow/cardiac troponin C transcriptional enhance^39^. FOS acts as a nuclear phosphoprotein that composes a close but non-covalently connected complex with JUN/AP-1 transcription factor. Both FOS and the JUN/AP-1 basic region appear to interact with symmetrical DNA half-sites in heterodimers. Multimeric SMAD3/SMAD4/JUN/FOS complexes are formed at the AP1/SMAD binding site during TGF-β activation to mediate TGF-β signaling. It has been documented that FOS critically functions in modifying the formation and maintenance of skeletal cell^40^ and it is believed FOS plays an significant role in signal switching, cellular proliferation and differentiation.

For further investigating functions of immune cells in PAH, CIBERSORT was performed for immune infiltration assays. Results displayed that NK cell activation, monocyte, T cell CD4 memory activation and mast cell infiltration were increased and T cell CD4 naive infiltration was decreased in peripheral blood of PAH patients contrasted with the control group, which may be related to the development and exacerbation of PAH. It is noteworthy that that it was monocyte that was the highest proportion in PAH peripheral blood. Previous researches showed that for coping with hypoxia, pulmonary monocytes could perceive hypoxia, permeate pulmonary arterioles, and accelerate vascular reforming, which suggested that the monocyte lineage functions directly in the morbidity of PAH^41^. NK cells activation is able to identify and eliminate cells infected with viruses or tumor lesions and degranulate perforin-containing granules and granzymes with the participation of FasL and TRAIL molecules^42^. Ormiston et al. demonstrated that NK cells in PAH patients generated a greater amount of matrix metalloproteinase 9, leading to deleterious effects on pulmonary vascular remodeling and functional impairment^43^. At most concomitant states, PAH is bound up with CD4 T cells defects and related to HIV or HHV-8 infection and some experimental studies have displayed that the application of NK cells and T cells suppressed the development of PAH^44^. In a clinical study, mast cells were abundantly found in PAH by quantitatively measuring the number and activity of mast cells in blood and urine among 44 PAH patients and 29 healthy individuals^45^. Besides, mast cells were discerned as connective tissue types expressing tryptase and chymotrypsin^46^. The above findings indicated that mast cells were conducive to the vascular pathophysiology of PAH, which is consistent with the findings of our study.

## Conclusion

In summary, results in present study manifested that the development of pulmonary hypertension probably is the result of imbalance between pulmonary vascular remodeling and immune micro environment. We discovered that genes SLC4A1, AHSP, ALAS2, CA1, HBD, SNCA, HBM, SELENBP1, SERPINE1, ITGA2B are the most notable markers of PAH. In addition, (TEAD4, TGIF2LY, GATA5, GATA1, GATA2, FOS) are predicted TFs regulating DEGs and may be regarded as potential targets for the prevention of pulmonary vascular restructure. Regulatory NK cell activation, monocyte, T cell CD4 memory activation and mast cell were detected as the most obvious immune cells infiltrating the peripheral blood of PAH. Since a large number of immune cells are clearly changed in PAH, although it is not clear how the immune system affects vascular remodeling, it can be conjectured that immune system goes a long way in this pathological process. The inadequacies of this study are that the insufficient amount of data may lead to results bias and yet we did not carry out trials to verify the results obtained. Further experiments on immune cells can identify targets to perfect immuno-modulatory therapy for PAH patients. To a certain extent, it could be interpreted like this, key genes and TFs are tightly related to the occurrence of PAH and regarded as underlying targets for therapeutic makers as well, however, it is necessary to explore the specific mechanism using some animal and cell experiments.

## Materials and methods

### Raw data acquisition and preprocessing

In the study, three gene expression profiles GSE22356, GSE131793 and GSE168905 were obtained from GEO (Home–GEO–NCBI (nih.gov)) database. GSE22356 was based on the GPL570 platform including 10 normal samples and 10 PAH samples. GSE131793 was based on the GPL6244 platform involving 10 healthy samples and 10 PAH samples. GSE168905 was based on the GPL16791 platform containing 9 control samples and 12 PAH samples. Raw data of these profiles were annotated according to their respective platform files following by probes switched into gene symbols, respectively^47^. The software Perl and R (version 4.0.2) were employed for data preprocessing.

### Identification of DEGs

For the purpose of identifying the significant DEGs between the PAH and the healthy group, Limma (linear models for microarray data) package was adopted processing three datasets with |log2 fold change (FC)|≥ 1 and adjusted p value < 0.05 as the thresholds^21^. We obtained the DEGs of the three datasets respectively. Additionally, volcano plots and heatmaps were generated to evaluate these selected DEGs.

### Functional and pathway enrichment analysis

To assess the biological functions of the total DEGs, we combined the DEGs for gene ontology (GO) annotation and Kyoto encyclopedia of genes and genomes (KEGG) pathway analysis applying online website DAVID (https://david.ncifcrf.gov/tools.jsp), which classifies gene functions into BP, CC and MF^48^. Then use R to generate bubble and bar charts to visualize the obtained results. P value < 0.05 was defined statistically significant.

### Protein–protein interaction (PPI) network and hub gene identification

PPI network among all DEGs was established based on an online tool STRING (https://cn.string-db.org/) and then software Cytoscape (version 3.7.2) was employed to adjust and visualize PPI networks^49^. Subsequently, we utilized a plug-in Cytohubba of Cytoscape to determine top 10 hub genes according to the MCC algorithms. All key genes’ scores were over 15 degrees.

### Construction of TF-DEG regulation network

For searching potential interactions between DEGs and transcription factors (TFs), another plugin iRegulon in Cytoscape used for the identification of DEG-targeted TFs. The enriched motifs in iRegulon were ranked depending on the direct targets by means of position weight matrix^50^. The TF-DEG crosstalk pairs were gained from databases such as TRANSFAC, TRED and so on. Then the TF-DEG regulatory network was visualized by Cytoscape.

### Immune cell infiltration analysis

Numerous studies have shown that PAH has a certain relationship with the immune micro environment, so we performed this analysis. The corrected gene matrix was uploaded to CIBERSORT website (CIBERSORT (stanford.edu)), a powerful online immune assay calculation tool, via a deconvolution algorithm to calculate the percentages of 22 kinds of infiltrating immune cells with P < 0.05 as standard^51^. Hereafter, the results of deduced immunocytes infiltration analysis were visualized by R language in different manners (Spplemetary Table 1).

## Supplementary Information

Supplementary Information.

## Data Availability

The gene expression profiles of GSE22356, GSE131793 and GSE168905 were downloaded from Gene Expression Omnibus (GEO) (https://www.ncbi.nlm.nih.gov/geo/query/acc.cgi?acc=GSE22356, https://www.ncbi.nlm.nih.gov/geo/query/acc.cgi?acc=GSE131793 and https://www.ncbi.nlm.nih.gov/geo/query/acc.cgi?acc=GSE168905).

## Abbreviations

PAH: Pulmonary arterial hypertension
RV: Right ventricular
DEG: Differentially expressed gene
KEGG: Kyoto encyclopedia of genes and genomes
TFs: Transcription factors
PPI: Protein–protein interaction
BP: Biological process
CC: Cell component
MF: Molecular function
PDE-5: Phosphodiesterase-5
ERAs: Endothelin receptor antagonists
TKI: Tyrosine kinase inhibition
ECM: Extracellular mechanism
TGF-β: Transcription growth factor beta
AE: Anion exchanger
GO: Gene ontology
GWAS: Enome-wide association study
NHLBI: American national heart, lung, and blood institute
cEVs: Cell-secreted extracellular vesicles
LC: Lung cancer
CAs: Carbonic anhydrases
ORF: Open reading frame
tPA: Tissue plasminogen activator
uPA: Urokinase
PAI-1: Plasminogen activator inhibitor 1 deficiency
EMT: Epithelial mesenchymal transition
