# Supplementary material for: Integrated bioinformatics analysis reveals marker genes and immune infiltration for pulmonary arterial hypertension
Source: Sci Rep. 2022 Jun 16;12:10154. doi: 10.1038/s41598-022-14307-6 (PMC9203517; doi:10.1038/s41598-022-14307-6)
Supplement: Supplementary file 1 — Supplementary Information. [file 41598_2022_14307_MOESM1_ESM.docx]

| Immune Cell Infiltration of PAH |
| --- |

| Input Sample | B cells naive | B cells memory | Plasma cells | T cells CD8 | T cells CD4 naive | T cells CD4 memory resting | T cells CD4 memory activated | T cells follicular helper | T cells regulatory (Tregs) | T cells gamma delta | NK cells resting | NK cells activated | Monocytes | Macrophages M0 | Macrophages M1 | Macrophages M2 | Dendritic cells resting | Dendritic cells activated | Mast cells resting | Mast cells activated | Eosinophils | Neutrophils |
| --- | --- | --- | --- | --- | --- | --- | --- | --- | --- | --- | --- | --- | --- | --- | --- | --- | --- | --- | --- | --- | --- | --- |

| GSM3819898 | 0.009943823 | 0.023682213 | 0.004978112 | 0.090924996 | 0.108537356 | 0.138232635 | 0.009962343 | 0 | 0 | 0 | 0.150586704 | 0 | 0.437845593 | 0 | 0 | 0 | 0 | 0.004941445 | 0 | 0.006753195 | 0.013611585 | 0 |
| --- | --- | --- | --- | --- | --- | --- | --- | --- | --- | --- | --- | --- | --- | --- | --- | --- | --- | --- | --- | --- | --- | --- |
| GSM3819900 | 0.008281158 | 0.019763979 | 0.004946802 | 0.124746838 | 0.090989427 | 0.12724691 | 0.007804126 | 0 | 0 | 0.002213909 | 0.142654829 | 0 | 0.441254011 | 0 | 0 | 0 | 0 | 0.005336178 | 0 | 0.004135816 | 0.011768769 | 0.008857247 |
| GSM3819902 | 0.00960084 | 0.029085584 | 0.005764429 | 0.09514336 | 0.098436872 | 0.12662017 | 0.008702538 | 0 | 0 | 0 | 0.153800704 | 0 | 0.436970105 | 0 | 0 | 0.010981044 | 0 | 0.005173419 | 0 | 0.007600843 | 0.003411681 | 0.008708412 |
| GSM3819904 | 0.009721793 | 0.032222923 | 0.003317809 | 0.099441254 | 0.116545311 | 0.123311283 | 0.004809515 | 0 | 0 | 0.017469325 | 0.134401296 | 0 | 0.427094099 | 0 | 0 | 0 | 0 | 0.006254701 | 0 | 0.01565723 | 0.009753462 | 0 |
| GSM3819906 | 0.004764308 | 0.029255671 | 0.0002 | 0.104030793 | 0.103345052 | 0.127433628 | 0.010396673 | 0 | 0 | 0 | 0.14977866 | 0 | 0.444629734 | 0 | 0 | 0.007057996 | 0 | 0.004926744 | 0 | 0.01134658 | 0 | 0.002828617 |
| GSM3819908 | 0.008030893 | 0.025126497 | 0.002971013 | 0.136097031 | 0.075506687 | 0.122484111 | 0 | 0 | 0 | 0.001573664 | 0.141204097 | 0 | 0.45732718 | 0 | 0 | 0 | 0 | 0.00572988 | 0 | 0.012124354 | 0.0039755 | 0.007849092 |
| GSM3819910 | 0.002209168 | 0.035150981 | 0.002078358 | 0.081347204 | 0.095707905 | 0.136277304 | 0.003969023 | 0 | 0 | 0.020892795 | 0.12867859 | 0 | 0.469401727 | 0 | 0.001 | 0 | 0 | 0.007542937 | 0 | 0.0128398 | 0.002933135 | 0 |
| GSM3819912 | 0.0007 | 0.028958854 | 0.004555998 | 0.104237627 | 0.108521534 | 0.11500903 | 0.005477829 | 0 | 0 | 0.021169202 | 0.150909358 | 0 | 0.405567203 | 0 | 0 | 0 | 0 | 0.00588108 | 0 | 0.011685415 | 0.019128132 | 0.018219103 |
| GSM3819914 | 0.008591915 | 0.021091407 | 0 | 0.115755708 | 0.091702856 | 0.14214306 | 0.008365735 | 0 | 0 | 0.010534295 | 0.130147836 | 0 | 0.44106864 | 0 | 0 | 0 | 0 | 0.004562806 | 0 | 0.007338551 | 0.009108628 | 0.009588562 |
| GSM3819916 | 0.018166868 | 0.037806207 | 0.0034843 | 0.085365829 | 0.10168045 | 0.151286998 | 0.012709145 | 0 | 0 | 0 | 0.139750841 | 0 | 0.421277447 | 0 | 0 | 0.009817342 | 0 | 0.004366936 | 0 | 0.003721794 | 0.010565842 | 0 |
| GSM5172150 | 0.008091034 | 0.027574546 | 0.003286736 | 0.094716055 | 0.106829713 | 0.125362448 | 0.004896081 | 0 | 0 | 0 | 0.155240881 | 0 | 0.435987647 | 0 | 0 | 0 | 0 | 0.005928287 | 0 | 0.016574397 | 0.013977115 | 0.001535061 |
| GSM5172151 | 0.004143347 | 0.033864245 | 0.002704261 | 0.089485509 | 0.107967192 | 0.115100354 | 0.00553303 | 0 | 0 | 0.025515902 | 0.137837817 | 0 | 0.450463301 | 0 | 0 | 0 | 0 | 0.005435117 | 0 | 0.012344739 | 0.009605187 | 0 |
| GSM5172152 | 0.007068668 | 0.022784413 | 0.005433884 | 0.098537993 | 0.111569776 | 0.125712945 | 0.006151436 | 0 | 0 | 0.005575085 | 0.14579219 | 0 | 0.445027418 | 0 | 0 | 0.001807784 | 0 | 0.005886772 | 0 | 0.008252042 | 0.010022152 | 0.0004 |
| GSM5172153 | 0.017684074 | 0.022825685 | 0.004984397 | 0.116955233 | 0.089985194 | 0.134215317 | 0.004101328 | 0 | 0 | 0 | 0.138143206 | 0 | 0.44208983 | 0 | 0 | 0.013879638 | 0 | 0.004904609 | 0 | 0.003904554 | 0.002951366 | 0.003375569 |
| GSM5172154 | 0.015328358 | 0.023683743 | 0.003708503 | 0.116300006 | 0.094589203 | 0.12130998 | 0.007031948 | 0 | 0 | 0 | 0.145433723 | 0 | 0.435117005 | 0 | 0 | 0.011779727 | 0 | 0.005224443 | 0 | 0.014415665 | 0.003025361 | 0.003052334 |
| GSM5172155 | 0.014583634 | 0.028498089 | 0.004017972 | 0.116239683 | 0.090146434 | 0.130459832 | 0.00519278 | 0 | 0 | 0 | 0.139063289 | 0 | 0.435473961 | 0 | 0 | 0.015056964 | 0 | 0.005068396 | 0 | 0.003640052 | 0.001855749 | 0.010703166 |
| GSM5172156 | 0.002859023 | 0.025464589 | 0.005067676 | 0.093849664 | 0.112258771 | 0.125293428 | 0.002928927 | 0 | 0 | 0.019243005 | 0.142205729 | 0 | 0.444044728 | 0 | 0 | 0 | 0 | 0.005539158 | 0 | 0.007984541 | 0.013192798 | 0.0001 |
| GSM5172157 | 0.003224217 | 0.026897808 | 0.005359556 | 0.092064942 | 0.105782415 | 0.119167435 | 0.003906282 | 0 | 0 | 0.029793121 | 0.135846775 | 0 | 0.455559842 | 0 | 0 | 0 | 0 | 0.005563604 | 0 | 0.006619107 | 0.010214897 | 0 |
| GSM5172158 | 0.018206791 | 0.020862965 | 0.003839541 | 0.124288648 | 0.08595041 | 0.1237657 | 0.004903472 | 0 | 0 | 0 | 0.140984717 | 0 | 0.43628099 | 0 | 0 | 0.010522681 | 0 | 0.005261251 | 0 | 0.013360716 | 0.001795793 | 0.009976324 |
| GSM556413 | 0 | 0.0007 | 0.0007 | 0.045650631 | 0.193093368 | 0.098526283 | 0.037350375 | 0 | 0 | 0.010545323 | 0.031659156 | 0 | 0.543904266 | 0 | 0 | 0 | 0 | 0.006287372 | 0.011843893 | 0 | 0.01123128 | 0.008495139 |
| GSM556414 | 0.045747087 | 0.041325857 | 0 | 0 | 0.165597895 | 0.26934686 | 0.014655796 | 0 | 0 | 0.085624503 | 0.080854633 | 0 | 0.245468249 | 0.025614305 | 0 | 0 | 0 | 0.006169696 | 0.019595119 | 0 | 0 | 0 |
| GSM556415 | 0.027268963 | 0.022009861 | 0.006076427 | 0.086402116 | 0.180209754 | 0.122312436 | 0.001511167 | 0 | 0 | 0.01376159 | 0.096710498 | 0 | 0.426285832 | 0.003881333 | 0 | 0 | 0 | 0.00275003 | 0.010819993 | 0 | 0 | 0 |
| GSM556416 | 0.022436832 | 0.034588139 | 0.006266297 | 0.093145516 | 0.104617658 | 0.15988324 | 0 | 0 | 0 | 0 | 0.208021812 | 0 | 0.357255401 | 0.003343734 | 0 | 0 | 0 | 0.001995877 | 0.008445494 | 0 | 0 | 0 |
| GSM556417 | 0 | 0.022069128 | 0 | 0.006941082 | 0.464868237 | 0.183476458 | 0.023637265 | 0 | 0 | 0 | 0.070805186 | 0 | 0.179937804 | 0.005278054 | 0 | 0 | 0 | 0.006971453 | 0 | 0 | 0.036015333 | 0 |
| GSM556418 | 0.04446242 | 0.02847544 | 0 | 0.070052071 | 0.096499474 | 0.290348027 | 0.010895156 | 0 | 0 | 0.021151122 | 0.132343658 | 0 | 0.280120669 | 0.001995386 | 0 | 0 | 0 | 0.004042744 | 0.014391856 | 0 | 0 | 0.005221977 |
| GSM556419 | 0 | 0.017856211 | 0 | 0.146573721 | 0.235487575 | 0.177769048 | 0 | 0 | 0 | 0.024635033 | 0.135786903 | 0 | 0.243607218 | 0 | 0 | 0.010796974 | 0 | 0.007487316 | 0 | 0 | 0 | 0 |
| GSM556420 | 0.018590969 | 0.021723937 | 0.004824542 | 0.077800747 | 0.190298912 | 0.110423663 | 0.037586782 | 0 | 0 | 0.042251866 | 0.143145739 | 0 | 0.291735198 | 0 | 0.001889535 | 0 | 0 | 0.009122285 | 0 | 0.026832368 | 0.023773458 | 0 |
| GSM556421 | 0 | 0.021532373 | 0 | 0.036132309 | 0.244451981 | 0.081138457 | 0.012961422 | 0 | 0 | 0 | 0.141230974 | 0 | 0.415146415 | 0 | 0 | 0.005577402 | 0.001944241 | 0.00634227 | 0 | 0.014945341 | 0.018596816 | 0 |
| GSM556422 | 0.003166993 | 0.001014892 | 0.007667179 | 0.234324091 | 0.146450786 | 0.01086027 | 0.008909286 | 0 | 0 | 0.079939057 | 0.148996417 | 0 | 0.344129567 | 0.001189125 | 0 | 0.001207849 | 0 | 0.001821191 | 0.010323297 | 0 | 0 | 0 |
| GSM3819897 | 0 | 0.034609682 | 0.004061611 | 0.052018138 | 0.110476149 | 0.139691716 | 0 | 0 | 0 | 0.042571621 | 0.124982975 | 0.001 | 0.465283986 | 0 | 0 | 0 | 0 | 0.005902483 | 0 | 0.012350621 | 0.007078259 | 0 |
| GSM3819899 | 0.018644691 | 0.024220319 | 0.001383236 | 0.121081483 | 0.094464764 | 0.11272026 | 0.002252703 | 0 | 0 | 0 | 0.163370137 | 0 | 0.430236036 | 0 | 0.0005 | 0.009938755 | 0 | 0.004357584 | 0 | 0.007903496 | 0.004213586 | 0.00474495 |
| GSM3819901 | 0.003375867 | 0.012238352 | 0.009297946 | 0.10648736 | 0.111869162 | 0.121848764 | 0.002504425 | 0 | 0 | 0.012651285 | 0.144189284 | 0 | 0.449696807 | 0 | 0 | 0 | 0 | 0.005294819 | 0 | 0.00807485 | 0.009733929 | 0.002737151 |
| GSM3819903 | 0.014733372 | 0.011676657 | 0.007779762 | 0.097637085 | 0.078270423 | 0.116836147 | 0 | 0 | 0 | 0.025999 | 0.135804979 | 0.004221719 | 0.46957845 | 0 | 0.002619535 | 0.020121613 | 0 | 0.005420208 | 0 | 0.004365266 | 0.001529615 | 0.00340617 |
| GSM3819905 | 0.022080271 | 0.023694747 | 0.004319672 | 0.111651941 | 0.09199452 | 0.136935367 | 0.006994259 | 0 | 0 | 0 | 0.154542822 | 0 | 0.411368675 | 0 | 0 | 0.0007 | 0 | 0.005404126 | 0 | 0.007647286 | 0.010090219 | 0.012539371 |
| GSM3819907 | 0.017169452 | 0.032268449 | 0.001 | 0.117069458 | 0.087459235 | 0.137132824 | 0.003670183 | 0 | 0 | 0 | 0.153791428 | 0 | 0.436259835 | 0 | 0 | 0.00248127 | 0 | 0.004144951 | 0 | 0.002724275 | 0.0004 | 0.004414409 |
| GSM3819909 | 0.003815083 | 0.018117143 | 0.006821358 | 0.095708825 | 0.093820347 | 0.145221716 | 0.0005 | 0 | 0 | 0 | 0.153087228 | 0 | 0.453778215 | 0 | 0 | 0 | 0 | 0.005930202 | 0 | 0.012232612 | 0.003429081 | 0.007508236 |
| GSM3819911 | 0.034230019 | 0.013593138 | 0.002244076 | 0.071268307 | 0.109779004 | 0.139572712 | 0.006021199 | 0 | 0 | 0.014208353 | 0.145915004 | 0 | 0.422784331 | 0 | 0 | 0.006813406 | 0 | 0.00628823 | 0 | 0.011132515 | 0.016149706 | 0 |
| GSM3819913 | 0.017713753 | 0.019054463 | 0 | 0.091220858 | 0.094485237 | 0.137192629 | 0.0012505 | 0 | 0 | 0 | 0.153813824 | 0 | 0.429568445 | 0 | 0 | 0.025575004 | 0 | 0.006091164 | 0 | 0.00548171 | 0 | 0.018552413 |
| GSM3819915 | 0.001627222 | 0.025269072 | 0.006516337 | 0.108418079 | 0.109172615 | 0.11347194 | 0 | 0 | 0 | 0.006242878 | 0.146730759 | 0 | 0.450023489 | 0 | 0 | 0.002507517 | 0 | 0.00590518 | 0 | 0.015153059 | 0.008961853 | 0 |
| GSM5172159 | 0.006992099 | 0.029403886 | 0.003574908 | 0.100094932 | 0.098339534 | 0.117386122 | 0.002530557 | 0 | 0 | 0.010626484 | 0.14689587 | 0 | 0.446941989 | 0 | 0 | 0 | 0 | 0.006048826 | 0 | 0.017182399 | 0.012973386 | 0.001009009 |
| GSM5172160 | 0.012099516 | 0.031247129 | 0.003668674 | 0.114542282 | 0.093939484 | 0.121729564 | 0.004874603 | 0 | 0 | 0 | 0.145803563 | 0 | 0.438932261 | 0 | 0 | 0.012875758 | 0 | 0.005281344 | 0 | 0.005017202 | 0.006436463 | 0.003552156 |
| GSM5172161 | 0.004017368 | 0.023595239 | 0.005575306 | 0.092930909 | 0.112744748 | 0.133386315 | 0.00553752 | 0 | 0 | 0.009223806 | 0.141256093 | 0 | 0.453238918 | 0 | 0 | 0 | 0 | 0.005680615 | 0 | 0.006933955 | 0.005879208 | 0 |
| GSM5172162 | 0 | 0.029333265 | 0.005386224 | 0.068162384 | 0.112869986 | 0.142829889 | 0.0007 | 0 | 0 | 0.018626143 | 0.135102374 | 0 | 0.453372085 | 0 | 0 | 0 | 0 | 0.006130852 | 0 | 0.020682067 | 0.006819569 | 0 |
| GSM5172163 | 0.011209998 | 0.021624161 | 0.005022232 | 0.088898243 | 0.107544244 | 0.132138477 | 0.004460422 | 0 | 0 | 0.01005338 | 0.145287757 | 0 | 0.453200075 | 0 | 0 | 0 | 0 | 0.005489519 | 0 | 0.010344568 | 0.004726925 | 0 |
| GSM5172164 | 0.006958407 | 0.020938585 | 0.00676551 | 0.07975125 | 0.106351394 | 0.128853701 | 0.004677829 | 0 | 0 | 0.03702224 | 0.12951444 | 0 | 0.45643217 | 0 | 0 | 0 | 0 | 0.006455212 | 0 | 0.006935564 | 0.009343698 | 0 |
| GSM5172165 | 0 | 0.028862845 | 0.005079069 | 0.082352043 | 0.112397391 | 0.137160434 | 0.004688562 | 0 | 0 | 0 | 0.152518389 | 0 | 0.460367095 | 0 | 0 | 0 | 0 | 0.00551908 | 0 | 0.007515941 | 0.003539151 | 0 |
| GSM5172166 | 0.002956289 | 0.03463208 | 0.002442289 | 0.087867245 | 0.110627128 | 0.125073444 | 0.006261886 | 0 | 0 | 0.002541872 | 0.153862621 | 0 | 0.448713893 | 0 | 0 | 0 | 0 | 0.005920939 | 0 | 0.01304745 | 0.006052864 | 0 |
| GSM5172167 | 0.006581928 | 0.019988786 | 0.006654507 | 0.096362461 | 0.110497052 | 0.131571744 | 0.004116785 | 0 | 0 | 0 | 0.150980481 | 0 | 0.448040655 | 0 | 0 | 0 | 0 | 0.005609744 | 0 | 0.008201984 | 0.009755252 | 0.001638622 |
| GSM5172168 | 0.0117942 | 0.019237176 | 0.005576061 | 0.093400741 | 0.109315809 | 0.12082601 | 0.003483702 | 0 | 0 | 0.019970693 | 0.139717483 | 0 | 0.444590769 | 0 | 0 | 0.0001 | 0 | 0.005926363 | 0 | 0.012344305 | 0.013250922 | 0.0005 |
| GSM5172169 | 0 | 0.028869716 | 0.005337872 | 0.095836147 | 0.116086417 | 0.122967706 | 0.00307687 | 0 | 0 | 0.009778264 | 0.144709531 | 0 | 0.448663657 | 0 | 0 | 0 | 0 | 0.005756673 | 0 | 0.011094287 | 0.00782286 | 0 |
| GSM5172170 | 0.014481682 | 0.019871992 | 0.006566887 | 0.110123046 | 0.095849775 | 0.125404501 | 0.005216982 | 0 | 0 | 0.005343318 | 0.141132981 | 0 | 0.432618497 | 0 | 0 | 0.018377872 | 0 | 0.005218761 | 0 | 0.003676846 | 0.004421657 | 0.011695201 |
| GSM556423 | 0.021549872 | 0 | 0 | 0.225707453 | 0.053442213 | 0 | 0.004873396 | 0 | 0 | 0.051752617 | 0.101317718 | 0.049610954 | 0.410051931 | 0 | 0 | 0.063067426 | 0 | 0.003717345 | 0.014518366 | 0 | 0.0004 | 0 |
| GSM556424 | 0 | 0.003478567 | 0.011224882 | 0.229477271 | 0.058027254 | 0 | 0.012647271 | 0 | 0 | 0.082523849 | 0 | 0.087483713 | 0.452315896 | 0.010016592 | 0.0003 | 0 | 0 | 0.006843654 | 0.030888709 | 0 | 0.014819395 | 0 |
| GSM556425 | 0 | 0.042437603 | 0.004371578 | 0 | 0.06882363 | 0.15476191 | 0.081623942 | 0 | 0 | 0.040689897 | 0.131685905 | 0.019047147 | 0.402634112 | 0.008217442 | 0 | 0 | 0 | 0.002890881 | 0.018828893 | 0 | 0.018632465 | 0.005354594 |
| GSM556426 | 0.106101625 | 0 | 0.005181707 | 0.159795981 | 0.028436526 | 0 | 0 | 0 | 0 | 0 | 0.143057643 | 0 | 0.510209543 | 0 | 0 | 0.030595865 | 0 | 0.002173303 | 0.014447808 | 0 | 0 | 0 |
| GSM556427 | 0 | 0 | 0.004586855 | 0 | 0.283115095 | 0.144592281 | 0.028442587 | 0 | 0 | 0 | 0.035011574 | 0 | 0.452068205 | 0 | 0.003638016 | 0.011638074 | 0 | 0.00811607 | 0.028791244 | 0 | 0 | 0 |
| GSM556428 | 0 | 0.016734581 | 0.005566803 | 0.011232375 | 0.005853874 | 0.176803195 | 0 | 0 | 0 | 0.054591605 | 0.019319407 | 0.009337899 | 0.302296664 | 0 | 0.0006 | 0 | 0.003222439 | 0.009117558 | 0 | 0.335939729 | 0.036190647 | 0.01322906 |
| GSM556429 | 0.053345187 | 0.045550652 | 0 | 0.107290174 | 0.003304708 | 0.064898543 | 0.018634003 | 0 | 0 | 0.046098056 | 0.048518226 | 0 | 0.553811254 | 0.01200027 | 0 | 0.003670917 | 0 | 0.00626035 | 0.036617661 | 0 | 0 | 0 |
| GSM556430 | 0.132016846 | 0 | 0 | 0.198555033 | 0 | 0.030177847 | 0 | 0.006850088 | 0.014346862 | 0 | 0.013864329 | 0.026928365 | 0.429205009 | 0 | 0.010171443 | 0.005026975 | 0 | 0.011507151 | 0 | 0.083422803 | 0 | 0.037927249 |
| GSM556431 | 0 | 0.032502971 | 0.0001 | 0.0003 | 0.070352125 | 0 | 0 | 0 | 0 | 0 | 0.285638337 | 0.007771741 | 0.547572807 | 0 | 0 | 0.01613658 | 0 | 0 | 0.008211849 | 0 | 0 | 0.031365868 |
| GSM556432 | 0 | 0.006657683 | 0.001569192 | 0.066448913 | 0.061849056 | 0 | 0 | 0 | 0 | 0.065804963 | 0.072901542 | 0 | 0.641337711 | 0 | 0 | 0 | 0 | 0.004708233 | 0.017662421 | 0 | 0 | 0.061060286 |
|  |  |  |  |  |  |  |  |  |  |  |  |  |  |  |  |  |  |  |  |  |  |  |
